# A theoretical perspective on solid-state ionic interfaces

**DOI:** 10.1098/rsta.2023.0313

**Published:** 2024-09-09

**Authors:** Javier Carrasco

**Affiliations:** ^1^ Centre for Cooperative Research on Alternative Energies (CIC energiGUNE), Basque Research and Technology Alliance (BRTA), Alava Technology Park, Albert Einstein 48, Vitoria-Gasteiz 01510, Spain; ^2^ IKERBASQUE, Basque Foundation for Science, Plaza Euskadi 5, Bilbao 48009, Spain

**Keywords:** solid electrolytes, ionic transport mechanisms, ionic conductors, interfacial dynamics, *ab initio* modelling, machine learning in materials science

## Abstract

Solid-state ionic conductors find application across various domains in materials science, particularly showcasing their significance in energy storage and conversion technologies. To effectively utilize these materials in high-performance electrochemical devices, a comprehensive understanding and precise control of charge carriers’ distribution and ionic mobility at interfaces are paramount. A major challenge lies in unravelling the atomic-level processes governing ion dynamics within intricate solid and interfacial structures, such as grain boundaries and heterophases. From a theoretical viewpoint, in this Perspective article, my focus is to offer an overview of the current comprehension of key aspects related to solid-state ionic interfaces, with a particular emphasis on solid electrolytes for batteries, while providing a personal critical assessment of recent research advancements. I begin by introducing fundamental concepts for understanding solid-state conductors, such as the classical diffusion model and chemical potential. Subsequently, I delve into the modelling of space-charge regions, which are pivotal for understanding the physicochemical origins of charge redistribution at electrified interfaces. Finally, I discuss modern computational methods, such as density functional theory and machine-learned potentials, which offer invaluable tools for gaining insights into the atomic-scale behaviour of solid-state ionic interfaces, including both ionic mobility and interfacial reactivity aspects.

This article is part of the theme issue ‘Celebrating the 15th anniversary of the Royal Society Newton International Fellowship’.

## 1. Introduction

My research trajectory has evolved from investigating ice nucleation on metal surfaces during my Newton Fellowship to exploring the intricate dynamics of ions within interfacial structures present in ion conductors for solid-state electrochemical devices. Throughout both endeavours, I extensively employed first-principle theoretical methods to elucidate the evolution of interfaces and the mechanisms governing diffusion processes at the atomic level. By shifting focus to ion conductors, I am delving into a field with pressing technological relevance. Here, understanding the atomic-level intricacies is paramount for enhancing various technologies, notably advancing battery performance and driving progress in energy storage solutions. This shift underscores a commitment to tackling both fundamental inquiries and practical challenges, reflecting a deepening comprehension of materials science and a dedication to pioneering innovation in areas of critical importance.

Solid-state electrochemical devices typically comprise two porous electrodes (the anode and the cathode) along with a dense solid electrolyte that separates these electrodes. The fundamental principle governing their electrochemical function is the distinctive behaviour of ionic conductors, where at least one ionic component remains relatively immobile while another exhibits high mobility. Pure ion-conducting solids excel as electrolyte materials, facilitating efficient ionic conduction and mechanical stability. Conversely, mixed conducting solids, characterized by significant electronic and ionic conductivities, play a versatile role. They can serve as effective electrodes and essential components for various related devices such as rechargeable batteries, fuel cells, chemical sensors or electrochromic windows.

Current first-principle methods based on density functional theory (DFT) calculations prove effective in computing electronic structures and ion mobility for relatively simple bulk materials [1,2]. And, in the realm of modelling solid-state ionic conductors with the necessary accuracy, DFT-based *ab initio* molecular dynamics (AIMD) simulations and nudged elastic band (NEB) methods emerge as the two most successful computational approaches [1–4]. However, their general use can be hindered by the associated high computational cost, particularly when attempting extensive exploration of the configurational space of complex systems. Identifying a configuration that accurately represents the lowest energy well in a complex potential energy surface (PES), characterized by numerous energy wells, is a complex task. This complexity arises from the significant dependence of the final optimized energy value on the starting point, which includes the coordinates of each constituent atom of the system [5]. Despite efforts to enhance these DFT-based approaches, such as leveraging crystal symmetry [6] or employing statistical techniques [7–9], the primary bottleneck remains the substantial computational time, especially when dealing with complex, low-symmetry systems like interfaces. And the systematic exploration of large compositional and configurational spaces, often encountered in the study of interfaces in applied materials [10], further exacerbates this limitation. Therefore, ion-conducting interfaces, arguably the most critical component of solid-state electrochemical devices, remain the least understood, necessitating a dedicated focus on overcoming the computational challenges associated with their modelling [11].

A promising new alternative arises in the form of machine-learned potentials (MLPs), akin to empirical interatomic potentials, enabling accurate and efficient computation of entire complex PESs. While many analytical forms of interatomic potentials have been proposed, the generation of high-accuracy potentials still heavily relies on human intuition and expertise. Machine learning methods can help to construct more versatile interatomic potentials than those based on simple fixed functional forms. Consequently, MLPs are proving highly valuable for large-scale systems, where other approaches may be unavailable or too inaccurate, and they are beginning to be applied to the computational intricacies associated with studying ion conductors [12,13].

In this perspective, recognizing the need for consistency and clarity, I aim to provide first an overview of some physicochemical principles underlying the theoretical description of solid-state ionic systems. To achieve this, I begin by introducing fundamental concepts in bulk solids, such as the classical diffusion model and chemical potential. These concepts serve as the building blocks for understanding how kinetics and thermodynamics manifests at the atomic level. Then, I delve into the modelling of space-charge regions (SCRs), pivotal in elucidating charge redistribution at interfaces involving solid-state conductors. SCRs emerge when materials with differing standard chemical potentials come into contact. Consequently, SCR models provide valuable insights into conceptualizing charge accumulation and depletion at interfaces, bridging the understanding between bulk materials and interfaces. This discussion sets the stage for exploring sophisticated atomistic-level computational tools, such as DFT and MLPs, which are essential for accurately capturing atomistic details relevant to reactivity and ionic transport at interfaces, including interfacial reactivity. Additionally, throughout this perspective, practical examples from the literature are provided as references, offering interested readers opportunities to delve deeper into the field of modelling solid-state ionic interfaces at the atomistic scale or connect with experimental work. This effort seeks to consolidate scattered data in the literature, presenting the topic in an accessible manner for newcomers eager to contribute to this evolving field.

## 2. Classical diffusion model and chemical potential in bulk solids

The current understanding of ionic diffusion in solid ionic conductors is rooted in the classical diffusion model. This model describes ionic transport as the hopping of single ionic carriers from one lattice site to another via interconnected diffusion paths within the crystal structural framework [14,15]. When considering structurally ordered materials, such as crystalline frameworks, the predominant mobile species are often point defects, such as ions occupying interstitial sites or vacancies in the lattice structure [16]. It is important to note that interstitial sites are typically unoccupied positions within the lattice, while vacancies represent empty spaces where lattice atoms are absent. Thus, the crystal’s structural framework dictates the energy landscape of ion migration. During ion diffusion, a mobile ion navigates this energy landscape, and the highest energy point along the diffusion path sets the energy barrier for ionic migration (*E*_m_). Moreover, the ionic conductivity (*σ*) is proportional to the concentration of mobile ion carriers (*n*_c_), such as vacancies or interstitials, multiplied by $exp\left(-E_{\text{a}}/k_{\text{B}}T\right)$ at temperature *T*, where *E*_a_ represents a characteristic activation energy for ion conduction. This activation energy encompasses not only $E_{m}$ but also the energy required to form the mobile defects ( $E^{f}$ ) if needed. Thus, achieving a high *σ* typically necessitates a low *E*_m_ and a high *n*_c_.

The classical diffusion model is not restricted exclusively to crystals with a perfect periodic structure but can also be extended to ionic carriers in aperiodic solids, including glassy materials. For example, as a basic approximation, one can introduce the concept of density of states of ionic carriers, ξ(ε) , where ε is the partial molar free energy for a specific ionic carrier [17]. While the density of states is not explicitly mentioned in subsequent sections, it serves as a fundamental concept that provides a more general framework for understanding diffusion in various types of solids. In the case of periodic crystals, ξ(ε) simplifies to a delta function, offering a straightforward description. For aperiodic solids such as glassy materials, ξ(ε) is usually represented by a Gaussian function, allowing for a more nuanced depiction of the energy landscape of ions. However, it should be noted that no universal model spans all types of glasses, as discussed in detail in [18].

Overall, the classical diffusion model is effective when applied to ionic conductors with well-defined structures and compositions. In these scenarios, where diffusion predominantly occurs through the bulk material, the classical model offers good predictions of diffusion behaviour. The model is also valuable under steady-state conditions, where concentration gradients of charge carriers remain constant over time, and at low to moderate temperatures, where thermal energy primarily drives atomic motion [19]. However, the model’s limitations and assumptions must be carefully considered, especially in complex systems. For example, it typically assumes uniform diffusion coefficients and neglects phenomena such as interfacial reactions and local variations in atomic structure. Furthermore, the classical diffusion model relies on Fick’s laws of diffusion, which assume linear relationships between concentration gradients and fluxes. However, in real-world scenarios, diffusion processes can deviate from these linear relationships. In particular, these deviations may involve lingering dynamical shifts resulting from the interaction between the hopping ions, fluctuating positions of other mobile charge carriers, and movements of the host structure [20–24]. The classical model may struggle to fully account for these complex non-Fickian behaviours.

An additional key aspect to rationalize the behaviour of charge carriers in solids near equilibrium is the concept of chemical potential, which is equal to the change in free energy of the solid upon the insertion of an additional ionic carrier or point defect. For a particular Fermi–Dirac distribution of $N_{\text{d}}$ ionic carriers within N possible crystallographic sites, the corresponding chemical potential within a small range of *ε* values, ∆ε , is expressed as:


(2.1) $$\mu \left(\Delta \varepsilon \right)=N_{d}\Delta \varepsilon -TS_{\text{config}}=\Delta \varepsilon +RT\ln m\left(\Delta \varepsilon \right)+RT\;\ln\frac{1}{1-m\left(\Delta \varepsilon \right)}.$$

Here, $S_{\text{config}}$ , *R* and *T* represent the configurational entropy ( kln⁡(NNd) ), the gas constant (product of Avogadro’s constant and the Boltzmann constant k ) and temperature, respectively. The term m(∆ε) denotes the mole fraction of defects, $N_{\text{d}}\left(\Delta \varepsilon \right)/N\left(\Delta \varepsilon \right)$ . The dependence of μ on the total charge carrier activity is expounded in detail in [17].

Under the assumption that the chemical potential depends solely on the mole fraction, with neglect of interactions among charge carriers (in a dilute defect concentration scenario), the chemical potential ( $\mu_{i}$ ) for ionic carrier *i* can be expressed as:


(2.2) $$\mu_{i}=\mu_{i}^{0}+\ln\frac{N_{d,i}}{N}.$$

Here, $\mu_{i}^{0}$ represents the standard chemical potential, encompassing concentration-independent energy and entropy terms such as enthalpy of formation or vibrational entropy.

At this juncture, we should acknowledge our access to chemical potentials as a foundational element for describing and comprehending the behaviour of ionic carriers in solids across various scenarios. These scenarios include the presence of ions at high concentrations associated with interfaces, as well as the interplay between mobile and immobile charge carriers within such regions. Subsequently, I utilize the concept of chemical potential as a starting point to construct models, aiming to theoretically address solid-state ionic interfaces at a first-principle level.

## 3. SCR at interfaces

Up to this point, the focus has been solely on ionic carriers within a homogeneous single phase. Yet, a strikingly different scenario unfolds when considering, for example, a blend of two immiscible coexisting solids, particularly when creating solid–solid interfaces through the juxtaposition of two distinct phases (forming a heterostructure) or through the encounter of differently oriented grains within the same compound (forming a grain boundary). The possibilities that arise in such instances are diverse.

**Figure 1.**
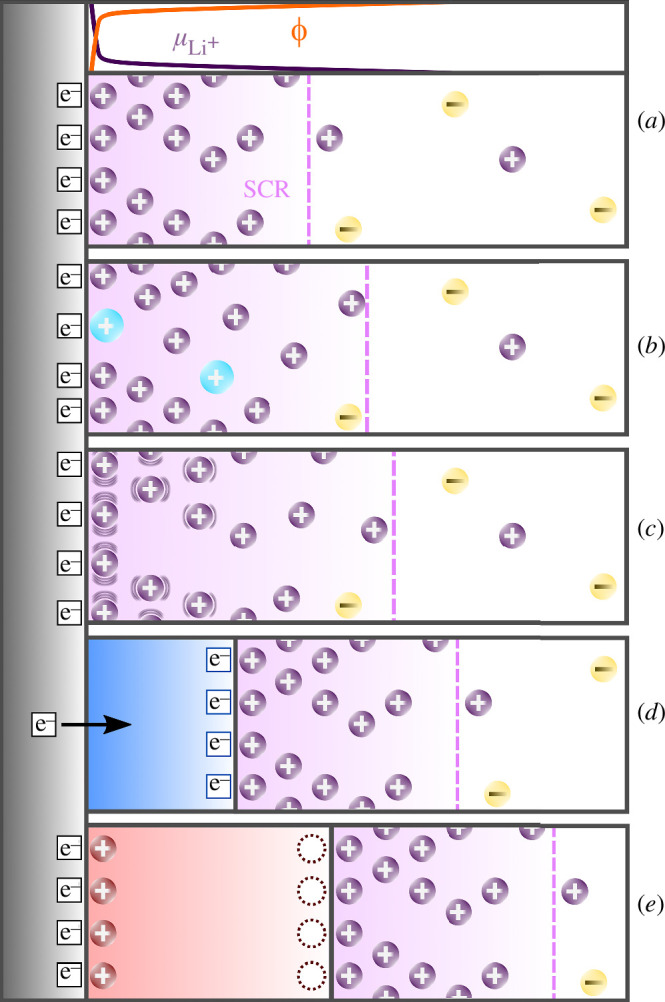
Schematic illustration of potential scenarios at the interface between an inert electrode (on the left), exhibiting electronic conductivity but lacking ionic conductivity, and an ionic conductor (on the right): (*a*) preservation of the atomic structure in the contact region, leading to the formation of an SCR (roughly delimited by the pink dashed line), illustrated alongside the schematic evolution (at the top) of chemical and electrostatic potentials perpendicular to the interface direction; (*b*) segregation of charged mobile impurities (light blue cations) from the ionic conductor to the interface; (*c*) structural rearrangements induced by elastic effects, depicted as oscillating waves surrounding atoms, might be particularly pronounced near the interface between the electrode and the ionic conductor; (*d*) emergence of an electronically conductive thin layer representing a third phase (in blue); and (*e*) development of an ionically conductive third phase (highlighted in red), accompanied by the possible formation of cationic vacancies at the interface with the ionic conductor, depicted by empty circles, which might introduce added complexity to the forming interface.

In the simplest scenario, the initial structure of the ionic conductor remains intact, and the interfacial region retains its atomic structure (figure 1*a*). However, the situation can become increasingly intricate (figure 1*b,c*). Close to the interface region, certain material parameters may undergo relatively smooth changes owing to structural rearrangements induced by gradient energy effects stemming from elastic influences. And, in cases of substantial interfacial misfit, more intricate defects, such as dislocations, can be generated.

Furthermore, the presence of impurities or other types of mobile charge species in one or both contacted phases can lead to the preferential accumulation or segregation of such species at the interface. In certain cases, entirely new phases may form due to chemical reactions between the two contacted phases (figure 1*d*,*e*). These additional interphases, resembling a passivation layer, might grow as a relatively thin layer between the two phases due to kinetic reasons. In particular, applied interests are interfaces between an electrode and a solid electrolyte in batteries [25], where the ion insertion into the electrode, accompanied by potential reactions, results in the depletion or enrichment of charge carriers near the interface depending on the electrode state of charge (i.e. operation voltage) [26–28].

In all these situations, a divergence in the standard chemical potential of ion carriers between the two contacted phases arises. Near the interface, charge carriers (ions and electrons) will be driven towards the material with the lowest standard chemical potential. If the ions or electrons are unable to migrate, a region in which charge builds up is created. In the simplest scenario, where the underlying atomic structures are preserved (figure 1*a*), the region of interfacial redistribution of ionic (and electronic) charge carriers is referred to as the SCR [29]. For an in-depth exploration of this matter, interested readers can refer to Maier’s review [30]. Notice that the formation of an SCR might indirectly influence ionic conductivity within the interface region. This impact primarily manifests through alterations in the charge carrier concentration perpendicular to the interface, which, as mentioned in §2, proportionally affects the ionic conductivity.

In SCR-based models, the total chemical potential is more conveniently expressed using an electrochemical potential:


(3.1) $${\tilde{\mu }}_{i^{z}}=\mu_{i^{z}}+ze\phi .$$

Here, z denotes the electrical charge of the mobile charge carrier *i*, e is the charge of an electron and ϕ corresponds to the electrostatic potential (conventionally referenced to zero when the system is electrically neutral).

To illustrate this concept, let us delve into the electrochemical charge redistribution occurring across the anode and an electronically insulating solid electrolyte interface in a solid-state battery. Consider a Li-based solid electrolyte where only Li^+^ ions are free to move. The driving force for charge redistribution is the imbalance between the Li^+^ electrochemical potentials across the interface, defined as the overpotential:


(3.2) $$\Delta {\tilde{\mu }}_{{\text{Li}}^{+}}={\tilde{\mu }}_{{\text{Li}}^{+}}^{\text{A}}-{\tilde{\mu }}_{{\text{Li}}^{+}}^{\text{SE}}.$$

Here, ${\tilde{\mu }}_{{\text{Li}}^{+}}^{\text{A}}$ and ${\tilde{\mu }}_{{\text{Li}}^{+}}^{\text{SE}}$ are the local electrochemical potentials of the anode and solid electrolyte phases adjacent to the anode–electrolyte interface. When ${\tilde{\mu }}_{{\text{Li}}^{+}}^{\text{SE}}$ is lower than ${\tilde{\mu }}_{{\text{Li}}^{+}}^{\text{A}}$ , Li^+^ ions experience a driving force to migrate from the anode to the electrolyte upon contact. This generates an electric field along the interface, eventually becoming large enough to counteract the chemical driving forces and halt the transfer of Li^+^ ions. Mathematically, equilibrium is reached when $\Delta {\tilde{\mu }}_{{\text{Li}}^{+}}=0$ . If $\Delta {\tilde{\mu }}_{{\text{Li}}^{+}}$ is negative, Li^+^ ions flow from the solid electrolyte into the anode, while the reverse occurs when $\Delta {\tilde{\mu }}_{{\text{Li}}^{+}}$ is positive. Moreover, we can derive the change in electrostatic potential across the interface by expressing the chemical potential of Li atoms in the anode phase in terms of Li^+^ and electron electrochemical potentials [2]:


(3.3) $$\mu_{\text{Li}}^{\text{A}}={\tilde{\mu }}_{{\text{Li}}^{+}}^{\text{A}}+{\tilde{\mu }}_{e^{-}}^{\text{A}}.$$

Then, by developing ${\tilde{\mu }}_{{\text{Li}}^{+}}^{\text{SE}}$ and ${\tilde{\mu }}_{e^{-}}^{\text{A}}$ according to equation (3.1) and considering $\Delta {\tilde{\mu }}_{Li^{+}}=0$ , we arrive at:


(3.4) $$\phi^{\text{SE}}-\phi^{\text{A}}=\frac{\mu_{\text{Li}}^{\text{A}}-\mu_{{\text{Li}}^{+}}^{\text{SE}}-\mu_{e^{-}}^{\text{A}}}{e}.$$

Here, $\phi^{\text{SE}}$ and $\phi^{\text{A}}$ are the electrostatic potentials for solid electrolyte bulk materials and anode, respectively. A similar expression can be derived to address the interface between the solid electrolyte and the cathode.

The application of continuum models to SCR [28,31,32] has revealed that the SCR typically has a thickness in the nanometre regime, consistent with experimental evidence [26,33]. The widths depend on applied potentials and the dielectric properties of the solid electrolyte, with thickness possibly reaching the order of a few ångströms for materials with small dielectric constants [31]. This implies that the SCR can occur over only a few atomic layers adjacent to the interface, as experimentally observed, for example, by Chen *et al*. [26], for Li_*x*_V_2_O_5_ electrodes in contact with Li_1.5_Al_0.5_Ge_1.5_(PO_3_)_4_ solid electrolyte. The limited thickness of the SCR restricts the applicability of continuum models, which are typically valid only for SCRs with widths above 10 Å [31], and confines their utility to describing qualitative features and providing rough estimations. This limitation stems from the fact that such models ignore the electronic structure of the solid electrolyte and neglect the description of discretized defect–defect interactions within the crystal lattice sites, which cannot be adequately described by bulk dielectric screening.

To appropriately model these aspects at the atomistic level, it is crucial to recognize that electrostatic potentials arising from the formation of an SCR have a profound impact on the creation of charged point defects because their presence directly influences the formation energy of such defects [34]:


(3.5) $$E_{i^{z}}^{\text{f,SCR}}=E_{i^{z}}^{\text{f,bulk}}+ze\phi .$$

Here, $E_{i^{z}}^{\text{f,bulk}}$ is the formation energy of a point defect $i^{z}$ at charge state *z* in the bulk of the material. According to standard defect theory [35], this can be obtained by computing total energies, atomic chemical potentials and Fermi levels (referred to as valence band maxima) using only first-principle calculations as input (e.g. DFT) [36–38]. Importantly, equation (3.5) implies that, for example, negatively charged defects cause $E_{i^{z}}^{\text{f,SCR}}$ to drop below $E_{i^{z}}^{\text{f,bulk}}$ . Therefore, defects that are unfavourable to form in the bulk might become stabilized at the SCR, as demonstrated by Dobhal *et al*. [27] in their investigation, the superionic conductor Li_10_GeP_2_S_12_ at the interface with a cathode.

In addition to determining net electrostatic potential drops across entire systems (i.e. anode/electrolyte/cathode), it is also possible to predict the spatial variation of charge carriers concentration within the SCR. To achieve this, Swift *et al*. [34] used the Poisson equation, assuming an infinite planar interface:


(3.6) $$\frac{\partial^{2}\phi }{\partial x^{2}}=\;-\frac{n_{c}^{+}\left(\phi \right)-n_{c}^{-}\left(\phi \right)}{\epsilon }.$$

Here, x represents the interface’s normal direction, $n_{c}^{\pm }\left(\phi \right)$ are the concentrations of charged carriers (neglecting free electrons under the assumption that the materials are effective electronic insulators) and ϵ is the dielectric constant of the material. Additionally, Swift *et al.* proposed to self-consistently combine this with a modified version of Fermi–Dirac defect distributions, considering that the space-charge concentration can reach very high values within a small region close to the interface. Consequently, the occupation of defect sites tends to approach full occupancy at high potential. However, such a scenario is deemed unphysical because defect–defect Coulomb interactions at high concentrations would significantly elevate the formation energy of defects. This outcome could potentially lead to material degradation through the formation of extended defects or decomposition into new phases. Alternatively, the system prefers to restrict the amount of defect at the SCR [28]. Therefore, the model incorporates an occupation fraction threshold as an *ad hoc* parameter into the Fermi–Dirac distribution to avoid complete saturation.

At this stage, it is crucial to recognize that the approaches discussed above solely rely on bulk properties for their formulations. Consequently, they can only accommodate average defect concentrations and electrostatic potential profiles. While we have seen that this averaging approach can provide valuable insights, as length scales approach those of the atomic lattice, more sophisticated models are needed to encompass defect–defect interactions [39]. In this regard, a promising avenue involves segmenting the interface region into layers, where each layer is distinguished by distinct defect formation energies derived from DFT calculations, contrasting with the consideration of only one average formation energy [40]. For example, such a layer-by-layer SCR model has been employed to ascertain oxygen vacancies and proton concentrations as well as electrostatic potentials in grain boundaries in BaZrO_3_ [41]. Concurrently, achieving access to more realistic interface geometries can be facilitated through interface structure prediction schemes, enabling the incorporation of interface reconstruction effects or simulating the impact of different surface terminations and compositions using lattice matching algorithms, as demonstrated, for example, by Gao and co-authors [36]. Moreover, the adoption of even more advanced methodologies, such as the Poisson–Cahn theory, holds the potential to precisely elucidate the electrical behaviour of complex interfaces and comprehensively characterize charge carriers across the entire concentration range at SCRs, ranging from dilute to concentrated [42].

## 4. Modelling of reactive interfaces

Up until now, all the models under consideration have operated on the assumption that the two phases in contact at the interface do not undergo chemical reactions. However, this assumption is not universally applicable. Solid electrolytes, for example, often possess narrow electrochemical windows, rendering them susceptible to reactions with electrode materials [43]. In reality, the final decomposition products may eventually give rise to an interphase between the solid electrolyte and the electrode (figure 1*d,e*). This interphase serves to passivate the solid electrolyte, inhibiting further decomposition. A notable example is the Li_2_PO_2_N solid electrolyte, which, upon contact with Li metal, decomposes into Li_3_N, Li_2_O and Li_3_PO_4_; remarkably, these decomposition products remain fully dense and form a stable, uniform passivation layer [44].

In principle, it is conceivable to extend SCR-based models discussed thus far to incorporate interfaces involving additional interphases with the electrodes and the pristine ionic conductor. This simply involves expanding the number of potential interfaces within the system and establishing the chemical and electrostatic potential profiles along each of them. However, the viability of such an approach hinges on having prior knowledge of potential decomposition reactions and products, which is often not readily available. In cases where such information is lacking, DFT-based simulations emerge as a well-suited method to scrutinize the thermodynamical stability of solid–solid contacts at the electrode–electrolyte interface [45,46]. This approach has demonstrated notable applicability in the exploration of crystalline compounds as coatings for numerous solid electrolytes in contact with cathode [47–51] and anode [52,53] materials.

It is also important to gain explicit insights into the dynamic evolution of electrochemical interfaces in both space and time. This involves obtaining access to metastable, short-lived intermediate reaction products, particularly considering that thermodynamically unfavourable interphase compounds may form under kinetic control [54–56]. Again, DFT calculations prove invaluable in this context, allowing probing interfacial reconstruction, space–charge effects and reactivity in general. This methodology is increasingly applied in the burgeoning field of solid-state batteries, particularly in the investigation of interfaces involving cathode materials or Li metal anodes in contact with inorganic solid electrolytes and potential protective coatings. For example, Lu *et al.* employed a combination of complementary experimental probes and DFT to examine the impact of cathodic Li content on the capacity fading of Li_*x*_CoO_2_/Li_10_GeP_2_S_12_ solid-state batteries [57]. Notably, the study unveiled that the interplay between SCR effects and interfacial side reactions ultimately governs the observed changes in cycling stability and capacity retention of these battery systems.

Similar approaches using DFT-based AIMD simulations have been applied to enhance our understanding of the interfacial reactivity of sulfide-based electrolytes at Li metal surfaces. These studies have contributed to characterizing decomposition products (e.g. Li_3_P, Li_2_S, LiCl and LiP) and unravelling underlying reaction mechanisms (e.g. stepwise breaking of P−S bonds and P−P bond recombination leading to the formation of reduced P_2_ and S^2−^ species) [54,58,59]. Such molecular-level insights are essential for the rational design of strategies to mitigate electrolyte decomposition, all while ensuring high (low) ionic (electronic) interface conductivity. In this regard, the combination of AIMD simulations with computational schemes adept at efficiently assessing interfacial ion diffusion [60,61] and electronic conductivity [62] holds significant promise. However, comprehending ion transport mechanisms in complex interfaces of solid-state ionic systems requires insight into global minimum energy paths (MEPs), which computationally poses a significant challenge. The global MEP delineates the most energetically favourable route for ion migration within a given PES. Although, in principle, AIMD simulations are designed to explore the entire dynamical landscape of a particular system with appropriate thermal weighting, they often encounter difficulties in escaping local MEPs that do not represent the global minimum [5]. This issue stems from inherent limitations in sampling efficiency, compounded by the complexity of the PES, resulting in computationally intensive simulation times required to adequately explore the configuration space. Additionally, it is relevant to acknowledge that when identifying MEPs for diffusing ions, the role of nearby charge carriers, derived from their concentration, must be considered. Consequently, regions of space with varying charge concentrations, driven by the formation of SCRs and resulting in concentration gradients perpendicular to the interface, may exhibit different MEPs. This variability occurs because changes in the local environment can affect ions’ preferences for various diffusion paths. Therefore, the likely dependence of MEPs on local charge carrier concentration adds complexity to sampling efforts.

To address the challenge of sampling efficiency, researchers have explored alternative strategies involving simplified interatomic potentials to approximate the complete PES. These potentials, typically expressed as parametric functions relating potential energy to atomic positions, enable the identification of MEPs through topological analysis [63]. Given a PES, identifying its critical points, representing stationary points with zero energy gradients, and gradient lines, delineating the steepest descent or ascent paths connecting critical points, play pivotal roles in this analysis. In this way, for example, by surveying crystalline Li- and Na-ion conductors in databases such as the Inorganic Crystal Structure Database (ICSD) [64], initial MEP approximations can be obtained. Subsequent DFT-based NEB calculations can refine these approximated MEPs, enhancing accuracy and maximizing computational resources [65]. For example, Wong *et al.* implemented the bond valence pathway analyser (BVPA) program, automating ion migration path discovery based on scanning bond valence energy landscapes (BVELs) [66]. Recent advancements have proposed replacing BVEL with electron density-based methods, which offer a more comprehensive representation of ion interactions [61,67]. These approaches facilitate the study of ion mobility in electrode–electrolyte interfaces and grain boundaries, significantly reducing computational time compared with stand-alone NEB-based schemes. They also open the door to exploring interface models and large supercells containing numerous symmetrically non-equivalent ion migration pathways. However, despite these improvements, certain steps in these methodologies still necessitate the computation of accurate energy profiles relying on costly DFT-based NEB calculations. Consequently, as the complexity of the modelled interfaces grows and DFT struggles to remain feasible within computational constraints, researchers are often limited to explicitly treating systems with only a few hundred atoms. In certain scenarios, this limitation can be alleviated by resorting to classical molecular dynamics [68–73]. For example, classical molecular dynamics has proven useful in understanding Li-ion diffusion at grain boundaries in oxide-type solid electrolytes, including Li_3_OCl [74] and Li_7_La_3_Zr_2_O_12_ [75]. Additionally, simplified approaches like the density functional tight-binding method have recently been employed to simulate the interfacial electronic properties of a Li|Li_2_PO_2_N|LiCoO_2_ solid-state battery at various states of charging [76]. However, these approaches face challenges due to their limited transferability, raising concerns about their straightforward application to different chemical systems or geometries than those for which they were parametrized. Therefore, AIMD remains preferable when applicable, as it possesses the inherent capability to capture detailed atomic interactions and dynamic behaviours, making it particularly suitable for scenarios involving intricate solid-state interfaces.

A recent and emerging alternative to conventional DFT-based simulations is the use of data-driven methods [12,77]. These methods aim to enhance conventional approaches by leveraging machine learning techniques, with the goal of significantly accelerating simulations while maintaining high accuracy. In the realm of solid-state ionic interfaces, a particularly noteworthy approach is the use of MLPs. These interatomic potentials essentially act as empirical force fields constructed with more versatile mathematical expressions than those based on simple fixed functional forms [13,78]. Developing accurate and transferable MLPs typically involves using first-principle data (usually obtained from DFT calculations) to train the underlying machine learning model. While this training procedure poses a significant computational bottleneck, given the need for large and diverse datasets, once an accurate MLP has been trained, it enables drastically accelerated simulations [79]. Currently, the most popular machine learning methods employed to construct MLPs for treating solid ionic conductors include neural networks (NNs) [80–83] and moment tensor potentials (MTPs) [84], as briefly presented in the following.

NNs draw inspiration from the intricate networks formed by biological neurons, adept at addressing nonlinear problems by mapping inputs onto a high-dimensional space. NNs achieve this by breaking down complex nonlinear functions into a series of linear transformations, characterized by parameters that can be learned. These parameters are linked by nonlinear activation functions, organized in layers. Specifically, in its simplest form, NNs consist of connected layers that linearly map an input vector $x\in R^{n_{\text{in}}}$ of dimension *n*_in_ to an output vector $y\in R^{n_{\text{out}}}$ of dimension *n*_out_ as:


(4.1) y=Wx+b.

Here, $W\in R^{n_{out}{\times n}_{in}}$ and $b\in R^{n_{out}}$ are parameters representing weights and biases, respectively. They are initially set randomly and then optimized. To introduce nonlinear relations, additional layers can be added to the NN along with nonlinear activation functions σ, resulting in h=σ(Wx+b) . Equation (4.1) can then be reformulated as:


(4.2) $$y=W_{L+1}h_{L}+b_{L+1}.$$

Here, $h_{L}$ is a hidden layer of the form:


(4.3) $$h_{L}=\sigma \left(W_{L}h_{L-1}+b_{L}\right).$$

A NN model can comprise L hidden layers, sequentially combined in what is known as a deep NN.

When NNs are employed in MLPs, they often rely on a locality assumption, restricting energy contributions to a certain distance from each atom [85]. This approximation is suitable for materials where short-range interactions dominate. However, in the case of ionic conductors, caution is warranted due to the non-negligible influence of long-range Coulomb interactions. Despite this consideration, NN-based MLPs demonstrate promising results, showing good agreement with experimental data and estimated errors comparable with those obtained through DFT-based AIMD simulations. For example, in the computation of Li vacancy diffusion in amorphous Li_3_PO_4_, NN-based MLPs exhibit excellent performance, achieving results comparable with DFT-based AIMD simulations with significantly reduced computational times: approximately four times faster [86]. In fact, NN potentials have found excellent applicability in studying ionic conductivity in solid-state electrolytes, as evidenced in investigations on Li_10_GeP_2_S_12_ [87], Li_7_La_3_Zr_2_O_12_ and Na_3_Zr_2_Si_2_PO_12_ [88].

Moving now to MTPs, this relatively novel class of MLPs relies on an efficient polynomial basis of interatomic distances and angles. In MLPs, the total energy of the system (*E* ^tot^) is defined as the sum of individual atomic contributions ( V ):


(4.4) $$E^{\text{tot}}=\sum_{i}^{N}V\left(s_{i}\right).$$

Here, N denotes the total number of atoms, and $s_{i}$ represents the set of atomic numbers and vectors $r_{ij}$ pointing from the central atom to its neighbouring atoms within a defined distance range. The central idea behind this approach is to approximate the atomic potentials $V(s_{i})$ through a linear combination of atom-centred symmetry basis functions ( $B_{\alpha }$ ), dependent on the local atomic environments:


(4.5) $$V\left(n_{i}\right)=\sum_{\alpha }\theta_{\alpha }B_{\alpha }\left(s_{i}\right).$$

Here, $\theta_{\alpha }$ denotes fitting parameters. The $B_{\alpha }$ functions consider the system’s symmetry and remain invariant under rotation, translation, reflection and permutation of chemically equivalent atoms. They are expressed as a contraction of moment tensor descriptors $M_{\mu ,\nu }$ :


(4.6) $$B_{\alpha }\left(n_{i}\right)=\;\prod_{i=1}^{\alpha k}M_{\mu ,\nu }\left(s_{i}\right),$$

which essentially involves combining the moment tensor indices through summation, reducing the rank of the tensor, and where


(4.7) $$M_{\mu ,\nu }\left(s_{i}\right)=\sum_{j}f_{\mu }\left(|r_{ij}|,\;a_{i},\;a_{j}\right)r_{ij}^{⨂\nu }.$$

Here, $a_{i}$ and $a_{j}$ represent the atomic types of the *i*th atom and its *j*th neighbour, respectively, and $r_{ij}$ is the position vector of the *j*th neighbour relative to the *i*th atom. The angular part is encoded by the outer product ⨂ of the $r_{ij}$ vectors, representing the Kronecker product of ν copies of the vector $r_{ij}$ (tensor of rank ν). The radial part of the atomic environment is given by the $f_{\mu }$ term, represented by a linear combination of polynomials:


(4.8) $$f_{\mu }=\;\sum_{\beta =1}^{N_{Q}}c_{\mu }^{\left(\beta \right)}Q^{\left(\beta \right)}\left(\left|r_{ij}\right|\right),$$

where


(4.9) $$\begin{array}{l} Q^{\left(\beta \right)}=\left\{ \begin{array}{ll} \varphi^{\left(\beta \right)}{\left(R_{max}-|r_{ij}|\right)}^{2}\;, & \left|r_{ij}\right|<\;R_{max}\\ 0 & \left|r_{ij}\right|\geq \;R_{max} \end{array}\right. \end{array}.$$

In equation (4.8), ${\{c}_{\mu }^{\left(\beta \right)}\}$ represents the set of radial fitting parameters defined for each pair of species and each μ , and $\varphi^{\left(\beta \right)}$ in equation (4.9) denotes Chebyshev polynomials on the interval $\left[R_{\text{min}},R_{\text{max}}\right]$ . During the machine learning training procedure, the parameters $\theta_{\alpha }$ , in equation (4.5), and $c_{\mu }^{\left(\beta \right)}$ are identified, aiming to minimize prediction errors for a set of target *ab initio*-derived variables, typically including energies, forces and stresses. The basis set’s form is determined based on the size of the radial basis $N_{Q}$ . The remarkable flexibility of the $B_{\alpha }$ functions in MTPs has found significant application in describing ionic mobility in complex materials, leading to a swift integration of this technique into the modelling toolkit for ionic conductors, as evident in recent studies on ionic diffusion in Na_3*−x*_Y_1*−x*_Zr_*x*_Cl_6_ [89] or high-entropy perovskite oxides [90].

While NN- and MTP-based MLPs have predominantly been utilized to investigate ionic diffusion in bulk ion conductors, their application to explicit solid-state ionic interfaces is rapidly gaining traction, offering access to long-time and large-scale simulations beyond the scope of DFT-based AIMD. For example, Jalem *et al.* employed MTP-based MLPs to explore amorphization effects at grain boundaries in β-Li_3_PS sulfide-type solid electrolytes, revealing significantly higher ionic conductivities at these interfaces compared with the bulk crystal by 1−2 orders of magnitude [91]. Similarly, Holekevi Chandrappa *et al*. developed an accurate MTP to investigate the S_8_/β-Li_3_PS_4_ interphase growth in a Li–S battery, uncovering rapid S_8_ decomposition and emphasizing the critical role of initial morphology in determining the interface thickness, subsequently influencing interfacial Li-ion diffusion pathways and their energy barriers [92]. Beyond gaining deeper insights into technologically relevant materials, MLPs can also be instrumental in screening promising new and doping-enhanced compounds for use as coating materials, showcasing low chemical reactivity and facilitating high Li-ion diffusion, as demonstrated by Wang *et al.* using both NN- [93] and MTP-based [94] MLPs.

## 5. Conclusion and outlook

**Figure 2.**
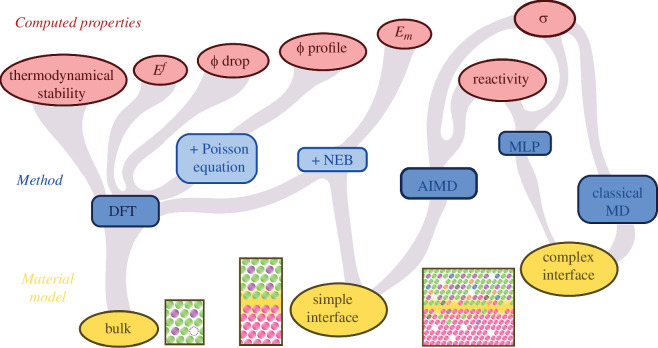
Overview of the main current combinations of material models and methodologies discussed in the text for assessing a diverse array of computable properties of solid-state ionic interfaces through first-principle calculations.

In the field of modelling and simulating solid-state ionic interfaces, the fusion of high-performance computing with DFT has undeniably ushered in an era where molecular-level exploration is approaching routine status. The insights garnered from this amalgamation extend beyond mere observation, providing a profound understanding of the intricacies inherent in solid-state ionic interfaces. From screening thermodynamically stable heterogeneous interfaces to identifying intermediate reaction products and assessing interfacial ionic conductivity, the current available methodologies have empowered researchers with a versatile toolkit (figure 2).

Envisioning the future, the trajectory of research suggests a shift towards an unprecedented paradigm in solid-state ionic interface modelling. This shift would manifest through the explicit inclusion of interphases formation and evolution in simulations, offering a more detailed understanding of the interfacial region. The evolving complexity of investigated interfaces necessitates accurate interatomic potentials, underscoring the importance of continued efforts to enhance model accuracy. Delving deeper, the focus on the first few atomic layers at interfacial regions emerges as a critical avenue for improvement. The limitations of continuum approaches become apparent as length scales approach those of the atomic lattice, necessitating more detailed models of defect–defect interactions to refine current understanding. Additionally, stressing the significance of the space-charge potential underscores the need for more sophisticated methodologies, like Poisson–Cahn theory, to precisely delineate the electrical behaviour of complex interfaces and comprehensively describe charge carriers across the entire concentration range at SCRs. At the same time, the prospect of protocols that include continuum-scale driving forces in atomistic simulations of explicit interfaces opens the door, for example, to full atomic-scale studies of the solid electrolyte interphase in batteries, offering a comprehensive exploration of the interfacial intricacies of applied materials of high technological interest. In tandem, the evaluation of electrochemical stability under applied voltage, involving simulations with an external electric field [95,96], adds a layer of sophistication to the research, providing insights into the dynamic behaviour of interfaces in practical scenarios. Yet, despite progress, challenges persist. DFT-based simulations of ionic conductivity are computationally demanding, especially for complex and reactive interfaces, leading to significant increases in time and cost. To address this challenge, researchers are exploring innovative solutions, including MLPs. MLPs hold promise for more accurately and efficiently navigating the complexities of solid-state ionic interfaces compared with traditional methods, particularly in identifying MEPs. However, their full potential hinges on overcoming hurdles like generating high-quality datasets and rigorous validations.

Additionally, the programme of work should encompass the validation of new developments in theory with well-defined experiments, enhancing the depth and applicability of the research. In essence, the integration of theory and experiments, coupled with a meticulous commitment to accuracy and a nuanced analysis of electronic structures, forms a triad that elevates the research endeavour. This holistic approach not only refines the theoretical frameworks but also ensures that the insights gained from simulations seamlessly align with the complexities of real-world materials and interfaces [11]. In this regard, the intricate measurements of actual chemical compositions and environments at interfaces, conducted through techniques such as Raman, solid-state nuclear magnetic resonance, and photoelectron spectroscopies, along with microscopic and diffraction techniques, provide invaluable real-world data. This wealth of experimental data becomes a touchstone for the predictions derived from first-principle modelling. The juxtaposition of theoretical predictions with empirical observations not only validates the accuracy of the models but also fosters a symbiotic relationship between theory and experimentation, driving the refinement of both.

In conclusion, as the field propels itself forward, the anticipated continued momentum of MLP applications heralds an era where the next generation of potentials will need to exhibit higher sophistication. This sophistication is crucial not only for representing electrified interfaces accurately but also for addressing the paramount importance of dataset quality in MLPs. It cannot be overstated that MLPs still rely profoundly on the accuracy of the training dataset. A poor-quality dataset will unequivocally lead to inaccurate results, thus underscoring the necessity for meticulous data curation and validation. Furthermore, this challenge highlights the imperative for continuous improvement in model development to mitigate the inherent risks associated with dataset limitations. Ultimately, confronting and surmounting these obstacles is indispensable for advancing the efficacy and reliability of MLPs, especially in the modelling of complex degradation processes. In this ongoing journey, the convergence of machine learning models and quantum mechanics calculations emerges as a beacon of progress [97], promising a future where the field achieves a harmonious synthesis of accuracy and efficiency. The intricate dance between theoretical frameworks and computational methodologies opens vistas of exploration, positioning the field at the forefront of transformative advancements in understanding and manipulating solid-state ionic interfaces.

## Data Availability

This article has no additional data.
